# Association of Primary Care Engagement in Value-Based Reform Programs With Health Services Outcomes: Participation and Synergies

**DOI:** 10.1001/jamahealthforum.2022.0005

**Published:** 2022-02-25

**Authors:** Julia Adler-Milstein, Ariel Linden, John M. Hollingsworth, Andrew M. Ryan

**Affiliations:** 1 Department of Medicine, University of California, San Francisco; 2 Center for Clinical Informatics and Improvement Research, University of California, San Francisco; 3 Department of Urology, University of Michigan, Ann Arbor; 4 Health Management and Policy, Center for Evaluating Health Reform, School of Public Health, University of Michigan, Ann Arbor

## Abstract

**Importance:**

Policy makers envision synergistic benefits from primary care reform programs that advance infrastructure and processes in the context of a supportive payment environment. However, these programs have been operationalized and implemented separately, raising the question of whether synergies are achieved.

**Objective:**

To evaluate associations between primary care engagement in voluntary delivery system and/or payment reform programs and health services outcomes.

**Design, Setting, and Participants:**

This was an observational longitudinal analysis of US ambulatory primary care organizations (PCOs) with attributed Medicare fee-for-service beneficiaries (1.6-1.9 million unique beneficiaries annually) using data for 2009, 2010, and 2015 to 2017; PCOs included multispecialty practices that delivered primary care. Data analyses were performed from January 2020 to December 2021.

**Exposures:**

Annual PCO participation in or recognition by (1) the Centers for Medicare & Medicaid’s meaningful use (MU) program, (2) the National Committee for Quality Assurance’s Patient-Centered Medical Home (PCMH) program, and/or (3) the Medicare Shared Savings Program (MSSP), an Accountable Care Organizations program.

**Main Outcomes and Measures:**

Independent and joint associations between an additional year of participation by a PCO in each of the 3 reform programs, and 3 types of outcomes: (1) hospital utilization (all-cause admissions, ambulatory care sensitive admissions, all-cause readmissions, all-cause emergency department visits); (2) evidence-based diabetes guideline adherence (≥1 annual glycated hemoglobin test, low-density lipoprotein cholesterol test, nephropathy screening, and eye examination); and (3) Medicare spending (total, acute inpatient, and skilled nursing facility).

**Results:**

The study sample comprised 47 880 unique PCOs (size ≤10 beneficiaries, 50%; ≤1-2 clinicians, 65%) and approximately 5.61 million unique Medicare beneficiaries (mean [SD] age, 71.4 [12.7] years; 3 207 568 [57.14%] women; 4 474 541 [79.71%] non-Hispanic White individuals) across the study years (2009, 2010, 2015-2017). Of the hospital utilization measures, only ambulatory care sensitive admission was associated with improved performance, showing a statistically significant marginal effect size for joint participation in MU and MSSP (−0.0002; 95% CI, −0.0005 to 0.0000) and MSSP alone (−0.0003; 95% CI, −0.0005 to −0.0001). For diabetes adherence, joint participation in PCMH and MU was associated with 0.06 more measures met (95% CI, 0.03 to 0.10) while participation in all 3 programs was associated with 0.05 more measures met (95% CI, 0.02 to 0.09). Stand-alone PCMH and stand-alone MU participation were also associated with improved performance. Joint participation in MU and MSSP was associated with $33.89 lower spending (95% CI, −$65.79 to −$1.99) as was stand-alone MSSP participation (−$37.04; 95% CI, −$65.73 to −$8.35).

**Conclusions and Relevance:**

This longitudinal observational study found that participation by PCOs in single or multiple reform programs was associated with better performance for only a subset of health services outcomes. More consistent and larger synergies may be realized with improved alignment of program requirements and goals.

## Key Points

**Question:**

Are there synergies between primary care organization participation in voluntary delivery system and payment reform programs (patient-centered medical home, meaningful use, and accountable care organization) and improved health services outcomes (hospital utilization, guideline adherence, and Medicare spending)?

**Findings:**

This observational longitudinal study of 47 880 primary care organizations including approximately 5.61 million Medicare beneficiaries found that participation in all 3 reform programs was associated with better performance for only diabetes guideline adherence. This outcome and 2 others (ambulatory care sensitive admissions and spending) also showed benefit from dual and single program participation.

**Meaning:**

These findings suggest that reform programs do not demonstrate consistent evidence of synergies, likely because program requirements and goals are not sufficiently aligned.

## Teaser

This was a longitudinal observational study of participation by primary care organizations in voluntary delivery and payment reform programs and its associations with hospital utilization, adherence to evidence-based care, and spending.

## Introduction

Given the central role of primary care in high-performing health care systems, much experimentation to improve health care quality and reduce spending has centered on transforming primary care practices.^1,2^ Some reform approaches focus on bolstering practice infrastructure. For example, the Centers for Medicare & Medicaid Services (CMS), through its Meaningful Use (MU) incentive program, requires that core primary care data on patient health issues, allergies, smoking status, and other elements, be documented in electronic health records (EHRs).^3^ Other approaches focus on medical practices’ processes that promote care coordination, a prime example being the National Committee for Quality Assurance’s Patient-Centered Medical Home (PCMH) recognition program.^4^ Other approaches, such as the Medicare Accountable Care Organizations (ACOs), focus on tying practice reimbursement to the value of care delivered.^5^

In concept, these primary care focused approaches should be synergistic, with investment in infrastructure and processes occurring in the context of a supportive payment model.^6^ Policy makers hoped for these synergies when they launched MU, the PCMH model, and ACOs, such as the Medicare Shared Savings Program (MSSP), but did not explicitly design these expectations into the programs.^7^ For example, key dimensions of the PCMH model, such as systematic tracking and follow-up of laboratory test results, are more likely to be effective when recorded in an EHR vs a paper-based patient chart. Moreover, effective use of new infrastructure and processes is likely intensified by increased financial rewards for better outcomes. Yet, these programs have been operationalized and implemented separately, raising the question of whether the intended synergies are achieved in terms of improved outcomes.

The literature on this critical question is limited. Individual programs have been extensively studied, generally suggesting that specific programs have achieved better outcomes, eg, lower spending associated with ACO participation.^8,9,10,11,12,13,14,15^ A small number of studies have found synergistic benefits from hospital participation in multiple reform programs.^16,17^ However, no prior work has examined the participation of primary care organizations (PCOs) in multiple programs over time to determine the extent to which these programs are working synergistically.^18^

Creating what is, to our knowledge, the first national longitudinal data set (2009, 2010, 2015-2017), this study sought to ascertain the relationship between PCO participation in 3 major voluntary reform programs—MU, PCMH, and MSSP—and various health services outcomes, assessing the independent and joint associations for each program. Although the programs changed during the study period and were partially combined under the Medicare Access and CHIP Reauthorization Act,^19^ our findings may be highly relevant to the open question of whether policy efforts should pursue more substantive alignment and integration or whether ongoing experimentation with more targeted but disconnected programs is effective for improving outcomes.

## Methods

This observational longitudinal analysis was reviewed and approved by the institutional review board of the University of California, San Francisco. Informed consent was waived because the analysis used only deidentified data. The study followed the Strengthening the Reporting of Observational Studies in Epidemiology (STROBE) reporting guidelines.

### Study Sample

The study samples included all Medicare fee-for-service beneficiaries in the standard Medicare 20% research identifiable files (Medicare Provider Analysis and Review, Outpatient, and Carrier Claims files) who were assigned to PCOs (identified by Taxpayer Identification Numbers [TINs]) in January to December of 2009, 2010, and 2015 to 2017. Specifically, we replicated the MSSP−ACO methodology for primary care attribution using the Medicare fee-for-service claims files.^20^ We identified beneficiaries enrolled in fee-for-service Medicare who received 1 or more primary care services from an eligible clinician, and we noted their corresponding TIN. Beneficiaries were then attributed to the TIN that provided the plurality (highest average cost) of their primary care during that calendar year. We followed this process for each of the 5 calendar years (2009, 2010, 2015-2017) for which we had Medicare claims files. We then limited the study sample to the 47 880 TINs that had 1 or more attributed beneficiaries in all 5 years to reflect the subset of organizations that were continuously eligible to participate in primary care reform programs. Therefore, the sample included primary care practices and multispecialty practices delivering primary care. We included this breadth because the programs under study applied to both types of practices. The final sample was at the beneficiary level and ranged from an annual total of 1.6 to 1.9 million unique beneficiaries.

### Study Measures

#### Outcomes

The study examined outcomes likely to be affected by PCO participation in each of the 3 programs. Only the ACO had an explicit set of outcomes built into its design.^21^ The MU and PCMH programs reward clinicians for processes that are likely to be associated with improved outcomes.^22,23^ We examined the requirements of each program and identified 3 outcomes that 2 or more programs were designed to target: (1) adherence to evidence-based practices; (2) avoidable hospital utilization (admissions/readmissions, ambulatory care sensitive [ACS] admissions, emergency department [ED] visits); and (3) spending (details are available in eTable 1 in the Supplement). Using the same Medicare fee-for-service claims, we constructed 4 hospital utilization outcomes: (1) rates of all-cause hospital admissions; (2) ACS hospital admissions (eMethods in the Supplement); (3) all-cause 30-day hospital readmissions; and (4) all-cause ED visits.

Clinical process quality was measured using 4 adherence-to-guideline measures based on the Healthcare Effectiveness Data and Information Set (HEDIS) for diabetes: at least 1 annual glycated hemoglobin test, low-density lipoprotein cholesterol test, nephropathy screening, and eye examination. We identified beneficiaries with diabetes based on the 2016 specifications,^24^ and then calculated results for each measure, as well as a composite score to reflect how many of the 4 measures were met. Individual measures were selected because they are broadly applicable to the Medicare population, can be measured in claims data, and have been used in related work.^25,26,27^ We examined the composite as a measure of overall adherence because no program was expected to affect 1 measure more than another.

The final measures included total annual Medicare spending determined by summing Medicare-paid amounts for a beneficiary’s care across all inpatient and outpatient files and adjusting for medical inflation to 2017 US dollars. We assessed 2 components of total spending—acute inpatient and skilled nursing facility—because reducing avoidable acute and postacute inpatient spending is a focus of ACOs and is enabled by capabilities in the 2 other programs.^14,27,28,29,30^

#### Participation in Primary Care Reforms

We measured TIN-level participation in each of the 3 programs at the calendar year level.^31^ We used the TINs as the organizational unit of program participation for 2 reasons: (1) MSSP participation is at the TIN level and, although MU and PCMH attestation occurs at the National Provider Identifier (NPI) level, both programs require substantial organizational investment, and therefore, individual NPI-level participation within larger organizations is rare; and (2) to identify organizations providing primary care to Medicare beneficiaries, we sought to replicate an existing definition based on Medicare claims to capture organizations that provide a plurality of primary care services to Medicare beneficiaries during multiple years. Because individual NPIs can switch among PCOs over time, this definition is logically operationalized at the TIN level.

We measured TIN-year level program participation using MU participation data from Medicare attestation files and Medicaid attestation files, PCMH recognition data, and MSSP participation data from the provider-level research identifiable file (eMethods in the Supplement). Longitudinal participation was then measured as a running sum of years of participation prior to and including a given year, allowing for noncontinuous years of participation. For example, for 2016 ACO participation, if a TIN had participated in 2014, 2015, and 2016, the value for 2016 would be 3.

#### Beneficiary and Organizational Characteristics

We adjusted for beneficiary-level factors that might confound the relationships between program participation and outcomes, including age, sex, and race and ethnicity (using definitions and categories from the Master Beneficiary Summary file), as well as the individual Elixhauser comorbidity categories^32^ for the given year (using all diagnoses in all files). To address heterogeneity across TINs, we adjusted for PCO characteristics (eg, size according to number and percentage of clinicians with a primary care, nonprimary care/medical, or surgical specialty) and characteristics of the county in which the PCO was located, per Medicare Data on Provider Practice and Specialty data, the Area Health Resource file, and the American Community Survey (described in our prior study).^31^

### Statistical Analysis

At the beneficiary-year level, the analytic data set linked beneficiaries to their attributed TIN in the given calendar year and TINs to the running sum of years of program participation in the given calendar year. For hospital utilization outcomes, we evaluated treatment effects using multilevel mixed-effects negative binomial regressions based on goodness-of-fit statistics.^33^ For diabetes adherence and spending, we used multilevel mixed-effects linear regressions. In all models, beneficiary, TIN, and community characteristics were specified as fixed effects. TINs were specified as the random intercept and in clustering standard errors. We used interaction terms between the main program variables (PCMH, MU, MSSP) to test the joint associations of an additional year of participation in each program for each outcome. After estimation, we computed marginal effect sizes for 1 year of program participation in individual programs and in all possible combinations (eg, 1 year of MU and MSSP) compared with no participation in any program. Treatment effects were considered statistically significant for *P* < .05; tests were 2-tailed.

Before selecting the analysis models, we tested the linearity assumption of each outcome as a function of the number of years of program participation, and we did not observe any nonlinear relationships (eFigure 1 in the Supplement). We also conducted several robustness tests that varied sample restrictions. Finally, we computed 3-year marginal effect estimates to assess whether multiple years of participation were associated with larger effect estimates. All data management and analyses were conducted using Stata, version 16 (StataCorp LLC). Analyses were conducted from January 2020 to December 2021.

## Results

### Study Sample Characteristics

The study sample comprised 47 880 unique PCOs (50% with 1-10 beneficiaries; 64.7% with 1-2 clinicians; 36.9% participated in MU for 3-6 years) and approximately 5.61 million unique Medicare beneficiaries (mean age [SD], 71.4 [12.7] years; 3 207 568 [57.14%] women; 4 474 541 [79.71%] non-Hispanic White individuals) across the study years (2009, 2010, 2015-2017). The most common comorbidities among the beneficiaries were uncomplicated hypertension (45.30%) and uncomplicated diabetes (21.43%). Additional key characteristics of the PCOs and the beneficiaries are shown in Tables 1 and 2, respectively.

**Table 1.** Characteristics of the Primary Care Organizations (PCOs) in the Sample

Characteristic	No. (%)
PCOs with unique TIN	47 880 (100)
Size^a^	
No. of attributed beneficiaries	
1-10	24 049 (50.23)
11-20	10 427 (21.78)
21-50	8680 (18.13)
51-99	2430 (5.08)
≥100	2294 (4.79)
No. of clinicians with unique NPI	
1-2	30 974 (64.69)
3-7	9590 (20.03)
8-12	2557 (5.34)
13-19	1436 (3.00)
20-99	2442 (5.10)
≥100	881 (1.84)
Clinician demographic information^a^	
Age, mean (SD), y	55.71 (9.52)
Female, mean (SD), %	29.54 (35.90)
Program participation, 2009-2017	
PCMH, y	
0	46 630 (97.39)
1-2	565 (1.18)
3-6	685 (1.43)
MU, y	
0	18 989 (39.66)
1-2	11 237 (23.47)
3-6	17 653 (36.87)
MSSP, y	
0	37 490 (78.30)
1-2	3366 (7.03)
3-6	7019 (14.66)

Abbreviations: MSSP, Medicare Shared Savings Program; MU, Meaningful Use program; NPI, National Provider Identifier; PCMH, National Committee for Quality Assurance’s Patient-Centered Medical Home recognition program; TIN, Taxpayer Identification Number.

^a^ Data for 2016, the year selected to report this subset of characteristics (variation among years was not notable).

**Table 2.** Characteristics of the Medicare Beneficiaries in the Sample

Characteristic	No. (%)
Observations (beneficiary-years)	10 632 689
Beneficiaries (unique participants)	5 613 525
Age, mean (SD), y	71.4 (12.7)
Sex	
Female	3 207 568 (57.1)
Male	2 405 957 (43.9)
Race and ethnicity^a^	
Black	558 546 (10.0)
Hispanic	299 762 (5.3)
Non-Hispanic White	4 474 541 (79.7)
Other	229 032 (4.1)
Unknown	51 644 (0.9)
Chronic conditions, relative frequency of most common	
Hypertension, uncomplicated	2 542 927 (45.3)
Diabetes, uncomplicated	1 202 978 (21.4)
Cardiac arrhythmias	779 157 (13.9)
Chronic pulmonary disease	707 866 (12.6)
Hypothyroidism	621 417 (11.1)
Outcomes, annual per beneficiary, mean (SD), count	
All-cause admissions	0.22 (0.70)
All-cause 30 d readmissions	0.11 (0.31)
Emergency department visits	0.42 (0.98)
Guideline adherence for 4 diabetes measures	2.24 (1.34)
Spending (2017 US dollars)	6679 (18 241)

^a^ Race and ethnicity data and categories are defined by the Master Beneficiary Summary File Race Code Base; categories were not aggregated to the “other” category.

Among the 3 programs, participation in MU was the highest, with 23.5% of PCOs having participated for 1 to 2 years, and an additional 36.9% for 3 to 6 years during our study period. (Table 1; eTable 2 in the Supplement). Participation in MSSPs was lower, with 7.0% having participated for 1 to 2 years, and an additional 14.7% for 3 to 6 years. Only 2.6% of PCOs had half or more of its clinicians recognized by PCMH; 1.2% had participated in the program for 1 to 2 years, and 1.4% for 3 to 6 years. The largest group for each of the 3 programs was composed of the PCOs that had not participated at all: MU (39.7%), MSSP (78.3%), and PCMH (97.4%).

### Model Findings

#### Hospital Utilization

**Figure 1.**
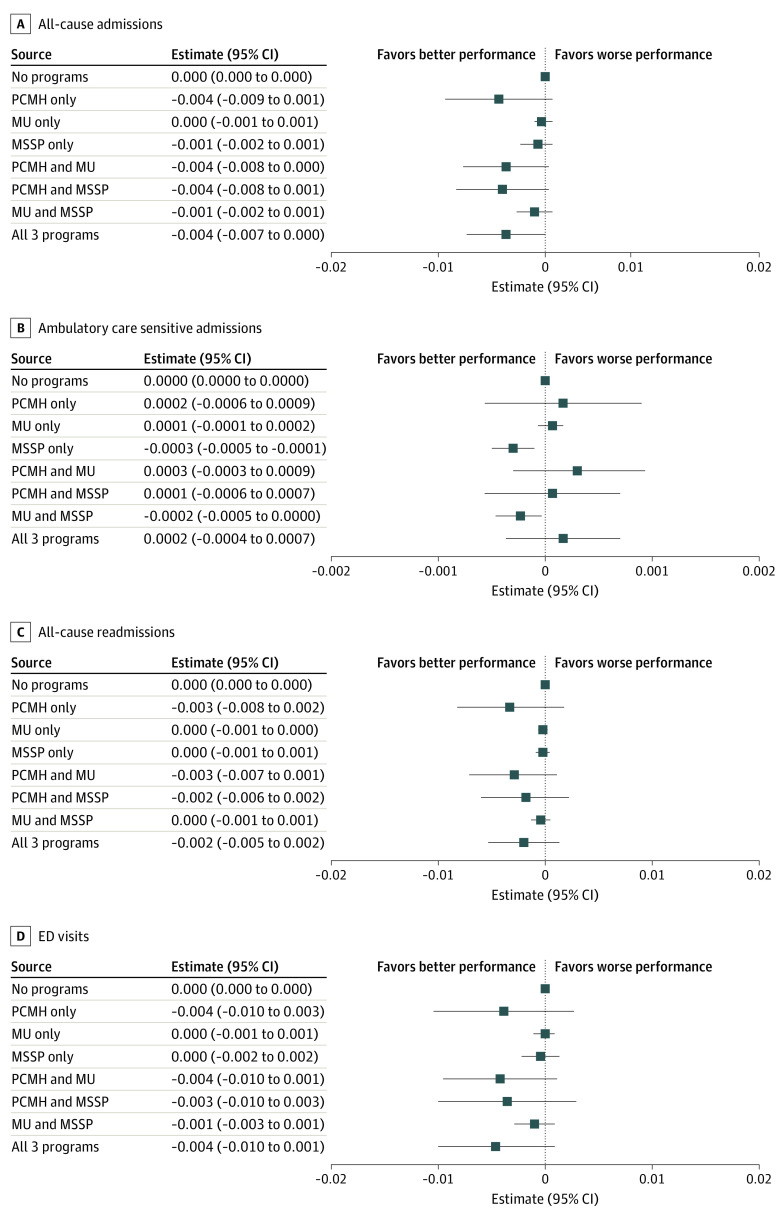
Marginal Effect Sizes of 1 Year of Program Participation Patterns: Hospital Utilization MSSP denotes Medicare Shared Savings Program; MU, Medicare & Medicaid Services’ Meaningful Use incentive program; and PCMH, National Committee for Quality Assurance’s Patient-Centered Medical Home recognition program.

There was no statistically significant marginal effect size for 1 year of program participation—not in 1 stand-alone program nor for any combination of the programs—for any measure of all-cause hospital utilization as compared with no program participation (Figure 1; eTable 3 in the Supplement). For ACS admissions, stand-alone MSSP participation was associated with a lower probability of ACS admission per beneficiary per year (−0.0003; 95% CI, −0.0005 to −0.0001; Figure 1; eTable 3 in the Supplement). Participation in both MU and MSSP was associated with a lower probability of ACS admission per beneficiary per year (−0.0002; 95% CI, −0.0005 to 0.0000).

#### Diabetes Guideline Adherence

**Figure 2.**
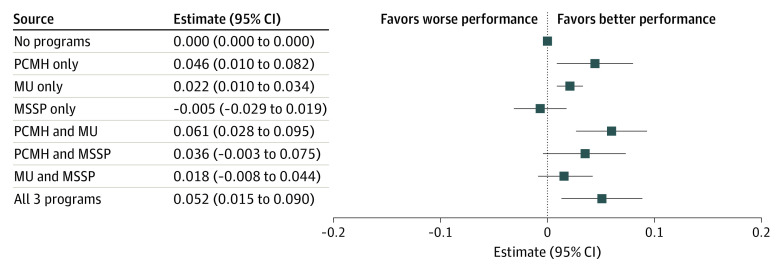
Marginal Effect Sizes of 1 Year of Program Participation Patterns: Adherence to Diabetes Evidence-Based Care Guidelines (Measures Met, *n* = 4) The 4 adherence-to-guideline measures based on the Healthcare Effectiveness Data and Information Set for diabetes is ≥1 each of the following: annual glycated hemoglobin test, low-density lipoprotein cholesterol test, nephropathy screening, and eye examination. MSSP denotes Medicare Shared Savings Program; MU, Medicare & Medicaid Services’ Meaningful Use incentive program; PCMH, and National Committee for Quality Assurance’s Patient-Centered Medical Home recognition program.

For the composite measure, stand-alone PCMH participation was associated with a 0.05 increase in the number of measures met of the 4 total measures (95% CI, 0.01 to 0.08; *P* = .01), and stand-alone MU participation was associated with an increase of 0.02 (95% CI, 0.01 to 0.03; *P* < .001; Figure 2; eTable 4 in the Supplement). Participation in both PCMH and MU was associated with an increased adherence of 0.06 (95% CI, 0.03 to 0.10; *P* < .001) and participation in all 3 programs increased adherence by 0.05 measures (95% CI, 0.02 to 0.09; *P* = .006). Participation in MSSP, alone or in a paired combination, was not associated with higher adherence. The mean adherence in the sample was 2.24 measures per beneficiary per year, such that these marginal effect sizes represent a 0.9% (for stand-alone MU, which had the smallest magnitude effect) to 2.7% (for PCMH−MU joint participation, which had the largest magnitude effect) improvement if all PCOs had participated for 1 year vs all not participating. For the individual measures, at least 3 of the 4 demonstrated a statistically significant positive 1-year marginal effect size consistent with the composite measure (supporting data provided in eTable 4 in the Supplement).

#### Annual Medicare Spending

**Figure 3.**
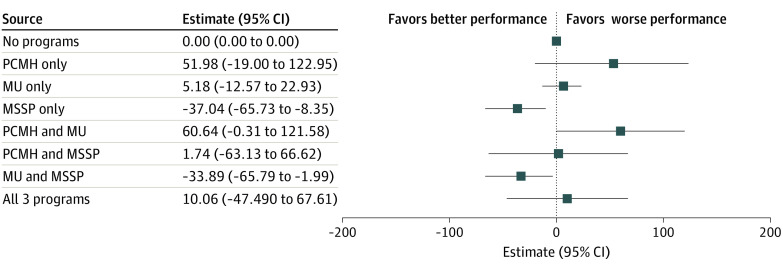
Marginal Effect Sizes of 1 Year of Varied Program Participation Patterns: Medicare Spending, $ MSSP denotes Medicare Shared Savings Program; MU, Medicare & Medicaid Services’ Meaningful Use incentive program; and PCMH, National Committee for Quality Assurance’s Patient-Centered Medical Home recognition program.

Stand-alone MSSP participation was associated with $37.04 lower spending per beneficiary per year (95% CI, −$65.73 to −$8.35; *P* = .01; Figure 3; eTable 5 in the Supplement). Participation in both MU and MSSP was associated $33.89 lower spending per beneficiary per year (95% CI, −$65.79 to −$1.99; *P* = .04). Mean annual spending per beneficiary was $6679.27; these marginal effect sizes represent savings of 0.51% (for MU−MSSP joint participation) and 0.55% (for stand-alone MSSP participation) if all the PCOs had participated for 1 year vs all not participating.

Skilled nursing facility spending aligned with total spending, with lower spending associated with stand-alone MSSP participation (−$9.07; 95% CI, −$15.53 to −$2.61; *P* = .006) and participation in both MU and MSSP (−$10.35; 95% CI, −$17.44 to −$3.26; *P* = .004; eTable 5 in the Supplement). For acute care spending, results were somewhat different, with lower spending associated with stand-alone MU participation (−$25.81; 95% CI, −$46.31 to −$15.20; *P* < .001), participation in PCMH and MSSP (−$57.32; 95% CI, −$105.11 to −$9.53; *P* = .02), participation in MU and MSSP (−$22.95; 95% CI, −$43.77 to −$2.13; *P* = .03), and participation in all 3 programs (−$58.94; 95% CI, −$100.80 to −$17.07; *P* = .006; eTable 5 and eFigures 2 and 3 in the Supplement).

### Robustness Tests

In robustness tests with alternative samples, results were consistent with primary estimates (eTable 6 in the Supplement). The 3-year marginal effect sizes were generally 3 times greater, and some marginal effect estimates for ED visits became newly statistically significant at the *P* < .05 level (supporting data reported in eTable 7 in the Supplement).

## Discussion

In this evaluation of national PCO participation in 3 voluntary reform programs, we found participation in all 3 programs to be low.^31^ However, this may not matter because an additional year of participation in all 3 programs was not consistently associated with improved outcomes. Although participation in all 3 programs was associated with modest increases in adherence to diabetes guidelines and lower acute care spending, single program participation was also associated with better performance, on these outcomes as well as others. Collectively, these findings suggest that there is no systematic program synergy, at least not with the outcomes examined. We suspect that this is because of their complex requirements and experimental nature, particularly MU and MSSP, which were novel in their designs and foci.

Indeed, prior work has pointed to the lack of aligned requirements as an important policy priority for decreasing participation burden and enhancing effectiveness.^34^ What remains unclear is how best to pursue this alignment. Although the present study did not reveal the specific mechanisms underlying how the programs do or do not interact to influence outcomes, there are some suggestive patterns. For diabetes guideline adherence, PCMH was the program—alone and in 2 combinations—that was associated with better performance. Similarly, for Medicare spending, MSSP was the program that—alone and in combination with MU—was associated with better performance. The analyses further suggest that spending reductions for these 2 patterns derived from lower ACS admissions as well as lower acute and skilled nursing facility spending. Given the lack of overlap among the programs’ incentive targets for participating PCOs, these results may reflect the primary focus of each program. For example, per beneficiary spending is tightly aligned to MSSP participation (and rewards), but it is not for either MU or PCMH. Thus, there may be a need to specifically align the goals with the participation requirements for each program.

Nonetheless, we did observe benefits from multiprogram participation, which is consistent with similar work in the hospital setting.^16,17^ A best case interpretation of these findings may be that the focus of any of the programs helps to realize benefits from a second program. For example, PCMH models, specifically the National Committee for Quality Assurance’s accreditation criteria, emphasize adherence to guidelines for chronic disease management, which may be reinforced by EHR workflows or alerts that are an option for meeting MU criteria.^35,36^ As another example, for the 3-year marginal effect estimates for all-cause ED visits, MU participation was present in all statistically significant combinations associated with lower utilization. Participation in MU may be associated with reduced ED utilization, particularly after multiple years of participation during which the progression of MU stages requires more advanced health information exchange. Specifically, having a robust EHR enables better health information exchange, such as admission-discharge-transfer notifications that alert primary care clinicians when their patient is in the ED. These alerts, in turn, allow the clinician to intervene in a timely manner, which can avoid a hospitalization^37^ and the associated costs. These relationships offer hypotheses that can be explored in future work to generate targeted guidance on how best to operationalize alignment of program goals and requirements.

For policy makers, these study findings should prompt reflection on the overall strategy to drive primary care practice transformation. Although program design has evolved, during and since the study period, federal programs have continued to struggle to achieve this alignment. In particular, programs separate uses of technology from processes involved in primary care transformation and accountable care. For example, at the programmatic level, CMS Innovation models have a category for accountable care and another category for primary care transformation in addition to several others. Even within the Merit-based Incentive Payment System, the health information technology component is separate from the other score components.^38^ Without more explicit efforts to synchronize EHR capabilities with the activities needed to deliver high-quality, low-cost primary care, it will be difficult for PCOs to realize substantial benefits from multiprogram participation. Similarly, there is a need to achieve greater incentive alignment between programs (extending to state/local Medicaid programs and private payor programs) and ensure that PCOs have a clear set of common spending and quality targets to work toward.

### Limitations

This study was limited because it included participation data from only 3 programs, thereby missing programs such as Pioneer ACOs and other PCMH programs. However, these other programs are either small or state-specific, such that the magnitude of potential bias is likely small. Similarly, it is possible that the PCOs that did not participate or stopped participating in a given program continued to meet the requirements, although this is unlikely given the financial penalties. In addition, the study’s Medicare claims data were limited to 5 years (2009, 2010, 2015-2017). Because we relied on these data to identify PCOs and measure outcomes, it is possible that some organizations would not have met our definition of a PCO or had systematically different outcomes in the intervening years (2011-2014), although we suspect either scenario is rare. In terms of outcomes, our diabetes-focused process measures may not generalize to other conditions, although the programs did not focus on any particular disease or condition. We also only captured outcomes for Medicare fee-for-service beneficiaries, which may not generalize to Medicare Advantage, dual eligible, or other beneficiary populations. Finally, these findings are observational because program participation was voluntary. Prior work has shown that estimates of the effect of ACOs can be biased by nonrandom attrition of patients and physicians.^27^ In addition, it is likely that PCOs with an interest in improving quality and resources to invest were more likely to participate. Despite taking several steps to improve the robustness of the study results—eg, using a large set of covariates to capture PCO characteristics and beneficiary demographic information—the associations we observed could be owed to unmeasured factors related to program participation and duration.

## Conclusions

This longitudinal observational study of PCOs during 9 years of participation in 3 large-scale delivery and payment reform programs failed to observe consistent synergistic benefits across outcomes compared with no program participation. These findings suggest that efforts to more explicitly bring program requirements and goals into alignment may produce greater gains through consistent synergistic benefits across a range of health services outcomes.

## Notes

Supplement 1. **eFigure 1.** Assessing Potential Nonlinear Relationships **eTable 1.** MU, PCMH, and ACO Program Components and Related Outcomes **eTable 2.** Detailed Program Participation **eTable 3.** One-Year Marginal Effect Sizes for Hospital Utilization **eTable 4.** One-Year Marginal Effect Sizes for Composite Measure and Individual Measures of Diabetes Guideline Adherence **eTable 5.** One-Year Marginal Effect Sizes for Total, Inpatient Acute, and SNF Medicare Spending **eFigure 2.** One-Year Marginal Effect Sizes for Medicare Spending (USD): Inpatient Acute **eFigure 3.** One-Year Marginal Effect Sizes for Medicare Spending (USD): Inpatient SNF **eTable 6.** Full Model Results and Robustness Tests **eTable 7.** One-Year vs Three-Year Marginal Effect Sizes
eMethods.
