# Supplementary material for: Association of Primary Care Engagement in Value-Based Reform Programs With Health Services Outcomes: Participation and Synergies
Source: JAMA Health Forum. 2022 Feb 25;3(2):e220005. doi: 10.1001/jamahealthforum.2022.0005 (PMC8903108; doi:10.1001/jamahealthforum.2022.0005)
Supplement: Supplement 1. — eFigure 1. Assessing Potential Nonlinear Relationships eTable 1. MU, PCMH, and ACO Program Components and Related Outcomes eTable 2. Detailed Program Participation eTable 3. One-Year Marginal Effect Sizes for Hospital Utilization eTable 4. One-Year Marginal Effect Sizes for Composite Measure and Individual Measures of Diabetes Guideline Adherence eTable 5. One-Year Marginal Effect Sizes for Total, Inpatient Acute, and SNF Medicare Spending eFigure 2. One-Year Marginal Effect Sizes for Medicare Spending (USD): Inpatient Acute eFigure 3. One-Year Marginal Effect Sizes for Medicare Spending (USD): Inpatient SNF eTable 6. Full Model Results and Robustness Tests eTable 7. One-Year vs Three-Year Marginal Effect Sizes eMethods. [file jamahealthforum-e220005-s001.pdf]

# Supplemental Online Content

Adler-Milstein J, Linden A, Hollingsworth JM, Ryan AM. Association of primary care engagement in value-based reform programs with health services outcomes: participation and synergies. *JAMA Health Forum.* 2022;3(2):e220005. doi:10.1001/jamahealthforum.2022.0005

**eFigure 1.** Assessing Potential Nonlinear Relationships

**eTable 1.** MU, PCMH, and ACO Program Components and Related Outcomes

**eTable 2.** Detailed Program Participation

**eTable 3.** One-Year Marginal Effect Sizes for Hospital Utilization

**eTable 4.** One-Year Marginal Effect Sizes for Composite Measure and Individual Measures of Diabetes Guideline Adherence

**eTable 5.** One-Year Marginal Effect Sizes for Total, Inpatient Acute, and SNF Medicare Spending

**eFigure 2.** One-Year Marginal Effect Sizes for Medicare Spending (USD): Inpatient Acute

**eFigure 3.** One-Year Marginal Effect Sizes for Medicare Spending (USD): Inpatient SNF

**eTable 6.** Full Model Results and Robustness Tests

**eTable 7.** One-Year vs Three-Year Marginal Effect Sizes

**eMethods.**

This supplemental material has been provided by the authors to give readers additional information about their work.

© 2022 Adler-Milstein JR, et al. *JAMA Health Forum*

**eFigure 1. Assessing Potential Non-Linear Relationships**

We tested the linearity assumption of each outcome as a function of the number of years of program participation for each program, and did not observe any non-linear relationships as reflected in the figures below. Larger CIs on PCMH due to smaller sample sizes.

Part A. Admissions

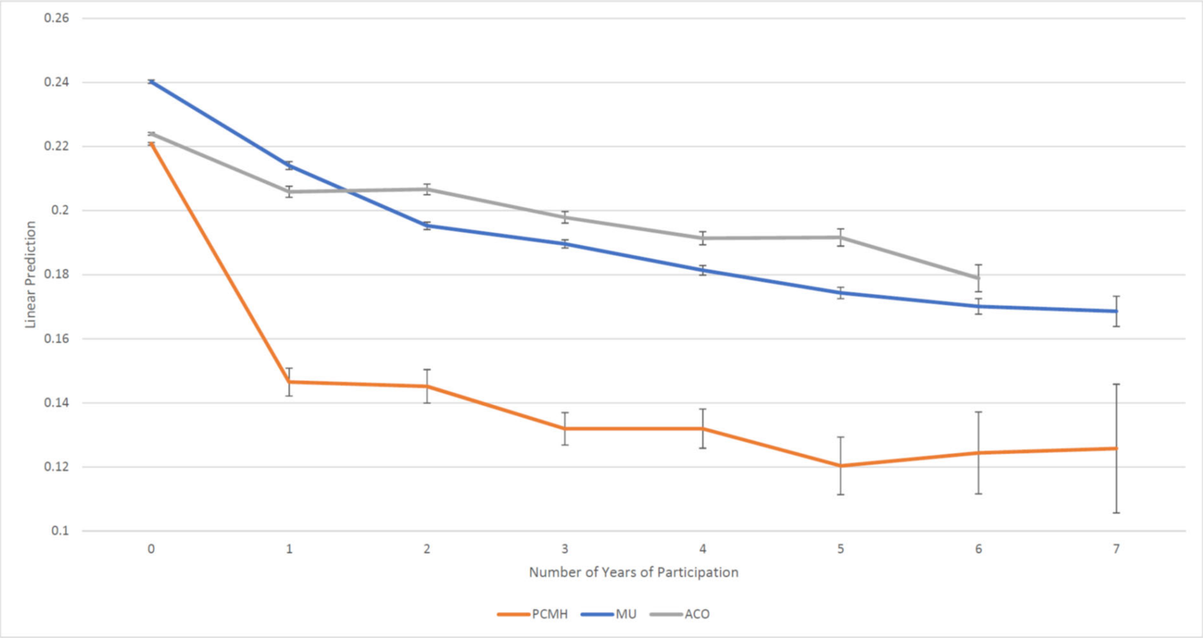


Part B. Readmissions

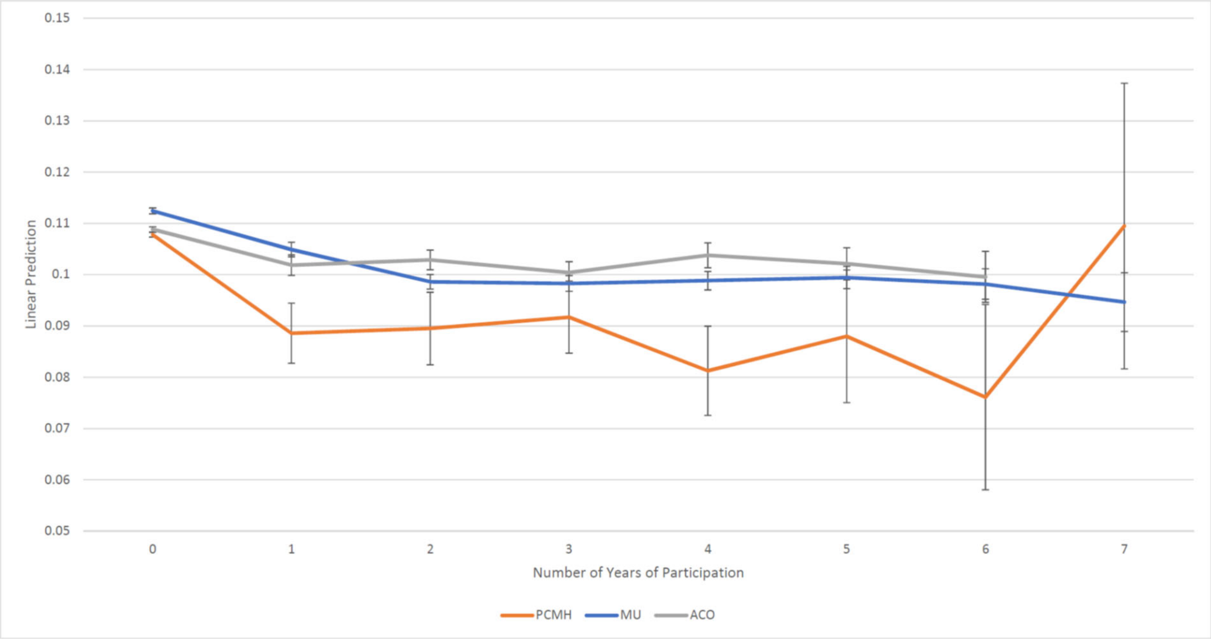


© 2022 Adler-Milstein JR, et al. *JAMA Health Forum*

Part C. ED Visits

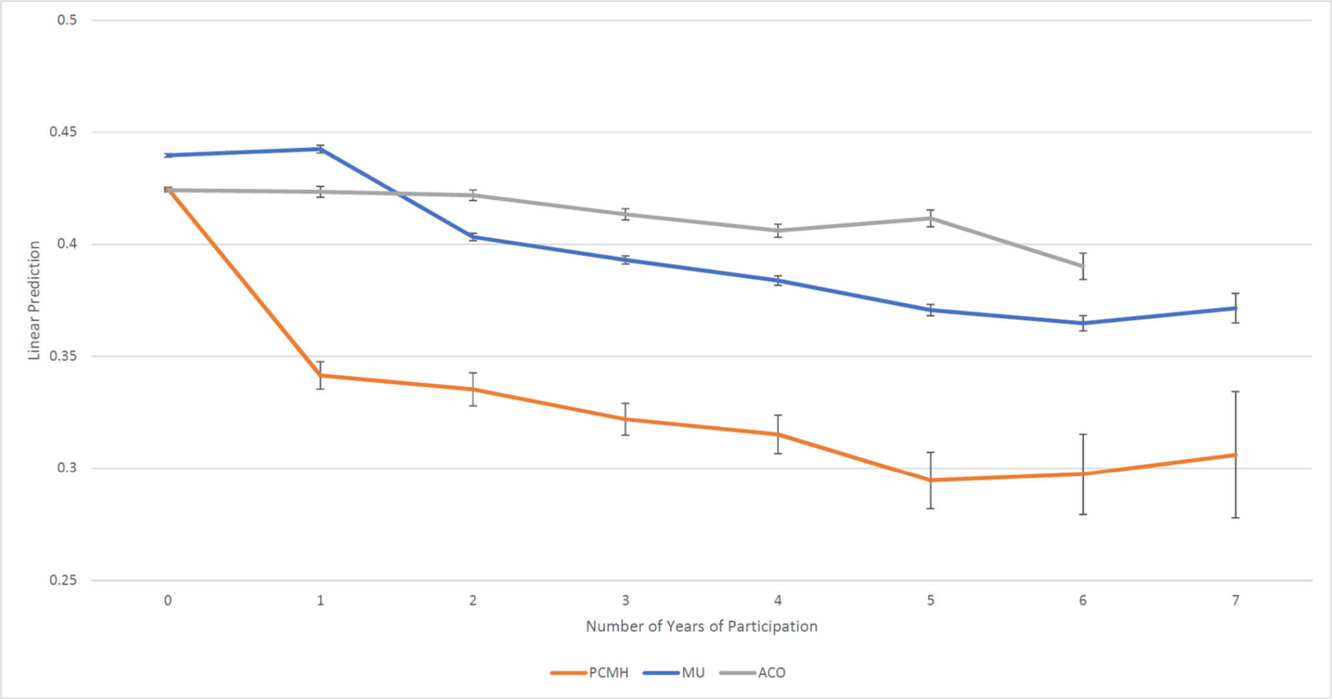


Part D. Diabetes Guideline Adherence

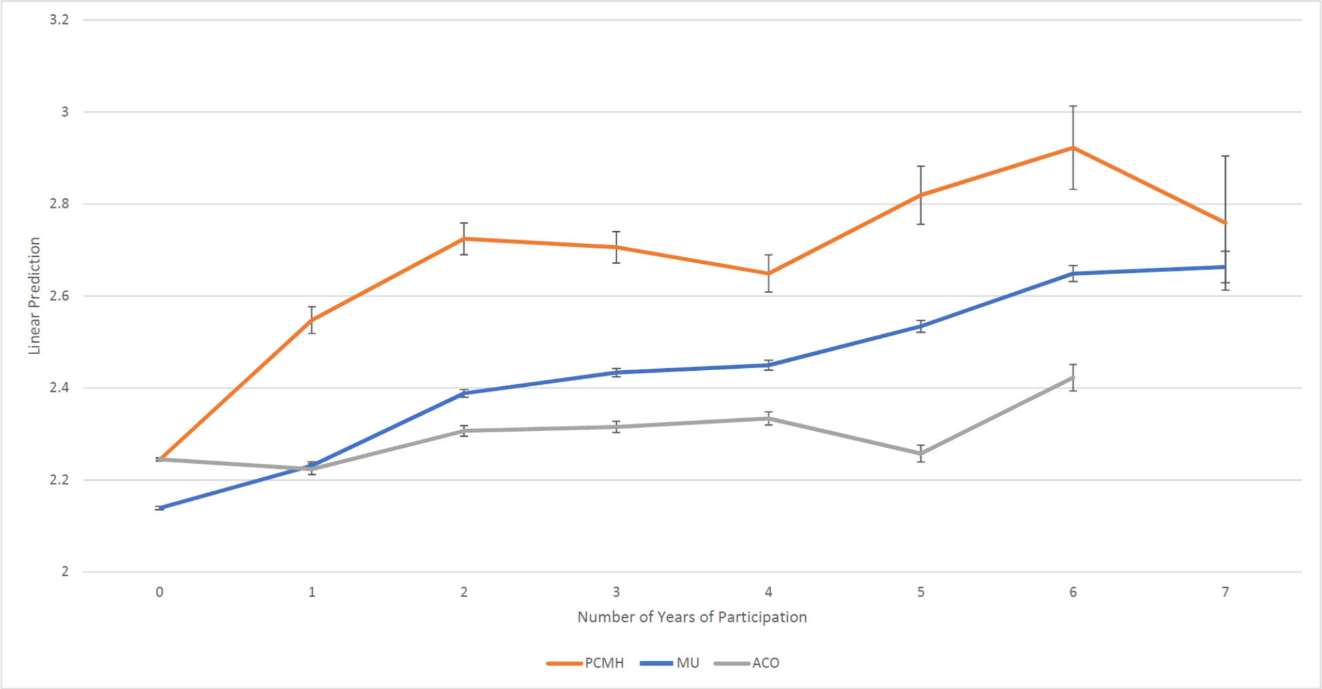


© 2022 Adler-Milstein JR, et al. *JAMA Health Forum*

Part E. Annual Spending

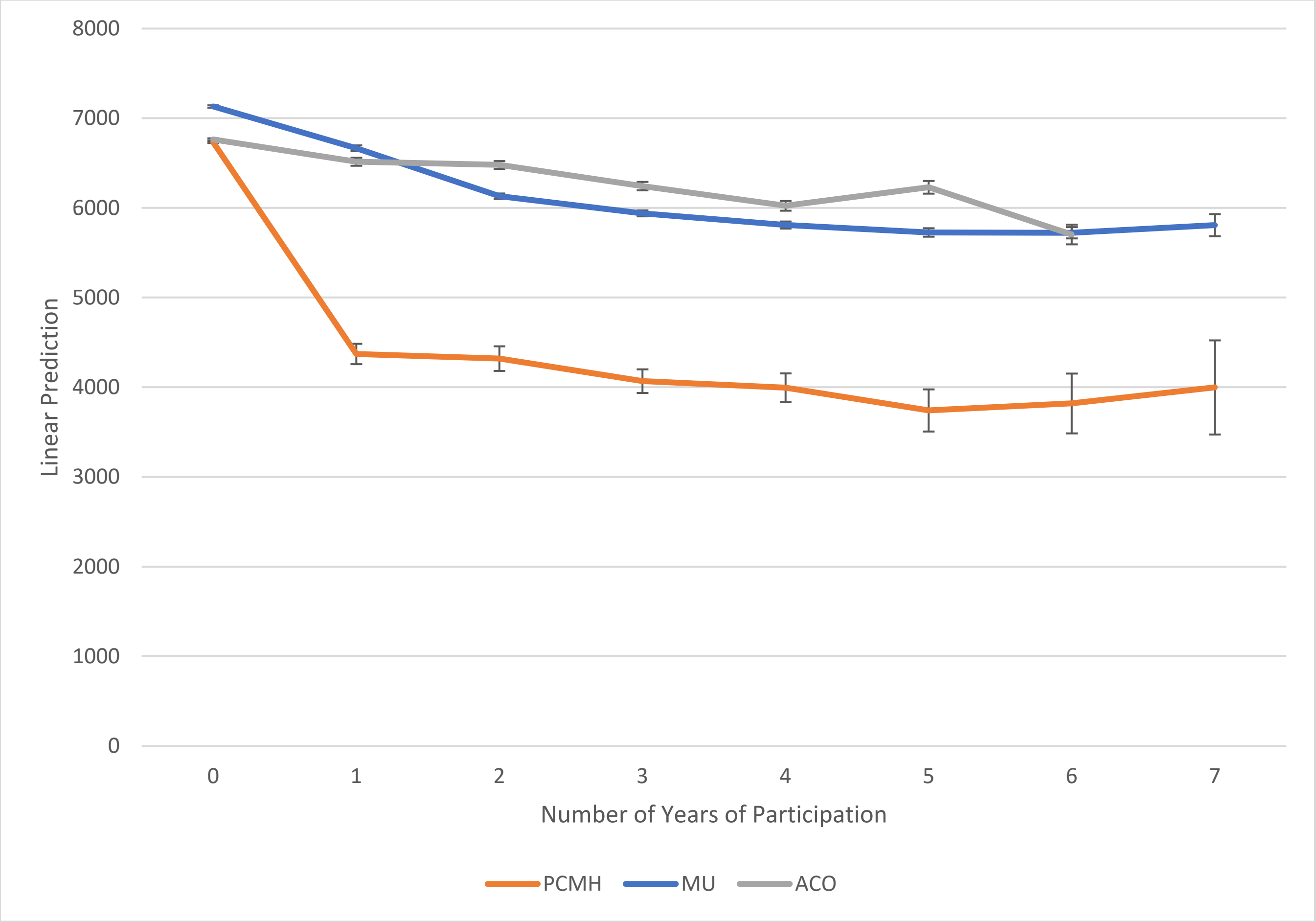


© 2022 Adler-Milstein JR, et al. *JAMA Health Forum*

| eTable 1. MU, PCMH, and ACO Program Components and Related Outcomes | | |
|---|---|---|
| **Reform Program** | **Program Components (Examples)** | **Related Outcomes** |
| Meaningful Use – Core Eligible Professional Criteria | (1) Capture basic demographic data including smoking status<br>(2) Computerized provider order entry for medications and ePrescribing<br>(3) Capture problem, medication, and medication allergy lists<br>(4) 1 clinical decision support rule<br>(5) Offer patient clinical summaries and access to information | √ Adherence to evidence-based practices<br>√ Spending |
| PCMH – NCQA<br>Must Pass Elements | (1) Access during office hours<br>(2) Use data for population management<br>(3) Care management<br>(4) Support self-care process<br>(5) Referral tracking and follow-up<br>(6) Implement continuous quality improvement | √ Adherence to evidence-based practices<br>√ Spending<br>√ Avoidable Hospital Utilization: Readmissions, ambulatory-care sensitive admissions, ED visits |
| ACOs – MSSP 33 quality measures | (1) Patient/Caregiver experience, including timely care, communication, patient education<br>(2) Care coordination/patient safety, including readmissions, ASC admissions, medication reconciliation, EHR incentive<br>(3) Preventive health, including immunizations, disease-specific and smoking<br>(4) At-risk populations, including diabetes, HTN, IVD, CAD, HF | √ Adherence to evidence-based practices<br>√ Spending<br>√ Avoidable Hospital Utilization: Readmissions, ambulatory-care sensitive admissions, ED visits |

© 2022 Adler-Milstein JR, et al. *JAMA Health Forum*

**eTable 2. Detailed Program Participation**

| **Program Participation** | |
|---|---|
| **Number of Organizations (TINs)** | 47,780 |
| **Years in PCMH** | % of orgs |
| 0 | 97.39 |
| 1 | 0.48 |
| 2 | 0.70 |
| 3 | 0.52 |
| 4 | 0.49 |
| 5 | 0.25 |
| 6 | 0.17 |
| | |
| **Years in MU** | % of orgs |
| 0 | 39.66 |
| 1 | 11.56 |
| 2 | 11.91 |
| 3 | 11.25 |
| 4 | 10.66 |
| 5 | 10.93 |
| 6 | 4.03 |
| | |
| **Years in ACO** | % of orgs |
| 0 | 78.3 |
| 1 | 4.28 |
| 2 | 2.75 |
| 3 | 4.22 |
| 4 | 3.95 |
| 5 | 3.20 |
| 6 | 3.29 |

© 2022 Adler-Milstein JR, et al. *JAMA Health Forum*

**eTable 3. One-Year Marginal Effect Sizes for Hospital Utilization**

| | **Probability of All-Cause Admission** | | | **Probability of ACS Admission** | | | **Probability of All-Cause Readmission** | | | **Probability of All-Cause ED Visit** | | |
|---|---|---|---|---|---|---|---|---|---|---|---|---|
| | Coeff | Lower 95% CI | Upper 95% CI | Coeff | Lower 95% CI | Upper 95% CI | Coeff | Lower 95% CI | Upper 95% CI | Coeff | Lower 95% CI | Upper 95% CI |
| No Programs | 0.000 | 0.000 | 0.000 | 0.0000 | 0.0000 | 0.0000 | 0.000 | 0.000 | 0.000 | 0.000 | 0.000 | 0.000 |
| PCMH Only | -0.004 | -0.009 | 0.001 | 0.0002 | -0.0006 | 0.0009 | -0.003 | -0.008 | 0.002 | -0.004 | -0.010 | 0.003 |
| MU Only | 0.000 | -0.001 | 0.001 | 0.0001 | -0.0001 | 0.0002 | 0.000 | -0.001 | 0.000 | 0.000 | -0.001 | 0.001 |
| MSSP Only | -0.001 | -0.002 | 0.001 | **-0.0003** | **-0.0005** | **-0.0001** | 0.000 | -0.001 | 0.001 | 0.000 | -0.002 | 0.002 |
| PCMH and MU | -0.004 | -0.008 | 0.000 | 0.0003 | -0.0003 | 0.0009 | -0.003 | -0.007 | 0.001 | -0.004 | -0.010 | 0.001 |
| PCMH and MSSP | -0.004 | -0.008 | 0.001 | 0.0001 | -0.0006 | 0.0007 | -0.002 | -0.006 | 0.002 | -0.003 | -0.010 | 0.003 |
| MU and MSSP | -0.001 | -0.002 | 0.001 | **-0.0002** | **-0.0005** | **0.0000** | 0.000 | -0.001 | 0.001 | -0.001 | -0.003 | 0.001 |
| All Three Programs | -0.004 | -0.007 | 0.000 | 0.0002 | -0.0004 | 0.0007 | -0.002 | -0.005 | 0.002 | -0.004 | -0.010 | 0.001 |

© 2022 Adler-Milstein JR, et al. *JAMA Health Forum*

**eTable 4. One-Year Marginal Effect Sizes for Composite Measure and Individual Measures of Diabetes Guideline Adherence**

| | **Count – 4 Measures** | | | **HbA1c Test** | | | **LDL-C Test** | | | **Nephropathy Screening** | | | **Eye Exam** | | |
|---|---|---|---|---|---|---|---|---|---|---|---|---|---|---|---|
| | Coeff | Lower 95% CI | Upper 95% CI | Coeff | Lower 95% CI | Upper 95% CI | Coeff | Lower 95% CI | Upper 95% CI | Coeff | Lower 95% CI | Upper 95% CI | Coeff | Lower 95% CI | Upper 95% CI |
| No Programs | 0.000 | 0.000 | 0.000 | 0.00 | 0.00 | 0.00 | 0.00 | 0.00 | 0.00 | 0.00 | 0.00 | 0.00 | 0.00 | 0.00 | 0.00 |
| PCMH Only | **0.046** | **0.010** | **0.082** | **0.01** | **0.00** | **0.02** | **0.05** | **0.00** | **0.11** | **0.13** | **0.08** | **0.18** | 0.03 | -0.01 | 0.07 |
| MU Only | **0.022** | **0.010** | **0.034** | **0.00** | **0.00** | **0.01** | **0.02** | **0.02** | **0.03** | **0.06** | **0.06** | **0.07** | **0.03** | **0.02** | **0.03** |
| MSSP Only | -0.005 | -0.029 | 0.019 | 0.00 | -0.01 | 0.01 | -0.03 | -0.05 | -0.02 | **0.01** | **0.00** | **0.02** | 0.00 | -0.01 | 0.01 |
| PCMH and MU | **0.061** | **0.028** | **0.095** | **0.02** | **0.00** | **0.03** | **0.07** | **0.02** | **0.11** | **0.18** | **0.14** | **0.22** | **0.05** | **0.02** | **0.08** |
| PCMH and MSSP | **0.036** | **-0.003** | **0.075** | **0.01** | **0.00** | **0.02** | 0.01 | -0.04 | 0.05 | **0.13** | **0.09** | **0.17** | **0.03** | **0.00** | **0.06** |
| MU and MSSP | 0.018 | -0.008 | 0.044 | 0.00 | -0.01 | 0.02 | -0.01 | -0.02 | 0.00 | **0.07** | **0.06** | **0.08** | **0.03** | **0.02** | **0.04** |
| All Three Programs | **0.052** | **0.015** | **0.090** | **0.01** | **0.00** | **0.03** | 0.02 | -0.02 | 0.06 | **0.17** | **0.14** | **0.21** | **0.05** | **0.03** | **0.08** |

© 2022 Adler-Milstein JR, et al. *JAMA Health Forum*

**eTable 5. One-Year Marginal Effect Sizes for Total, Inpatient Acute, and SNF Medicare Spending**

| | Total | | | | Inpatient: ACUTE | | | | Inpatient: SNF | | | |
|---|---|---|---|---|---|---|---|---|---|---|---|---|
| | Coeff | P-value | Lower 95% CI | Upper 95% CI | Coeff | P-value | Lower 95% CI | Upper 95% CI | Coeff | P-value | Lower 95% CI | Upper 95% CI |
| No Programs | 0.00 | 0.00 | 0.00 | 0.00 | 0.00 | 0.00 | 0.00 | 0.00 | 0.00 | 0.00 | 0.00 | 0.00 |
| PCMH Only | 51.98 | 0.15 | -19.00 | 122.95 | -36.65 | 0.22 | -95.61 | 22.30 | 14.44 | 0.16 | -5.62 | 34.51 |
| MU Only | 5.18 | 0.57 | -12.57 | 22.93 | **-25.81** | **<0.001** | **-36.41** | **-15.20** | -2.13 | 0.24 | -5.71 | 1.45 |
| MSSP Only | **-37.04** | **0.01** | **-65.73** | **-8.35** | 3.43 | 0.75 | -17.23 | 24.08 | **-9.07** | **0.006** | **-15.53** | **-2.61** |
| PCMH and MU | 60.64 | 0.05 | -0.31 | 121.58 | **-57.32** | **0.02** | **-105.11** | **-9.53** | 9.92 | 0.25 | -6.81 | 26.64 |
| PCMH and MSSP | 1.74 | 0.96 | -63.13 | 66.62 | -38.13 | 0.14 | -88.16 | 11.90 | -2.85 | 0.78 | -22.40 | 16.70 |
| MU and MSSP | **-33.89** | **0.04** | **-65.79** | **-1.99** | **-22.95** | **0.03** | **-43.77** | **-2.13** | **-10.35** | **0.004** | **-17.44** | **-3.26** |
| All Three Programs | 10.06 | 0.73 | -47.49 | 67.61 | **-58.94** | **0.006** | **-100.80** | **-17.07** | -4.98 | 0.56 | -21.72 | 11.76 |

© 2022 Adler-Milstein JR, et al. *JAMA Health Forum*

**eFigure 2. One-Year Marginal Effect Sizes for Medicare Spending (USD): Inpatient Acute**

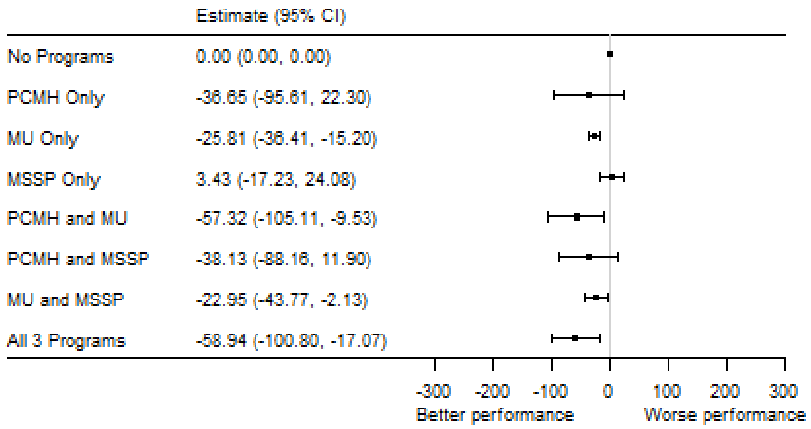


**eFigure 3. One-Year Marginal Effect Sizes for Medicare Spending (USD): Inpatient SNF**

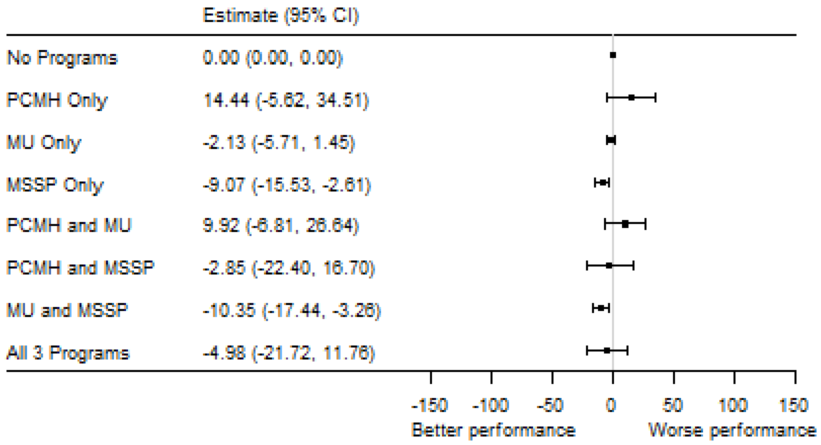


© 2022 Adler-Milstein JR, et al. *JAMA Health Forum*

**eTable 6. Full Model Results and Robustness Tests**

*Since the AHRF data are not available for communities with less than 20,000 residents (thereby excluding 2,507 TINs), we re-estimated models without these community characteristics in our full sample (47,880 TINs). We also re-estimated models with TINs that had at least 20 assigned beneficiaries (thereby limiting the sample to 29,475 TINs), in order to assess whether any associations dissipated due to the potential for undue influence of variability from small practices. All models include TIN, beneficiary, and calendar year controls (as listed in Tables 1 and 2)*

***Panel A***

| | **All Cause Admits** | | | | | |
|---|---|---|---|---|---|---|
| | **2017 with AHRF** | | **2017 without AHRF** | | **2017 >= 20 benes** | |
| | Coeff | P-Value | Coeff | P-Value | Coeff | P-Value |
| # years in PCMH | -0.016 | .11 | -0.011 | .29 | -0.021 | .05 |
| # years in MU | 0.000 | .93 | 0.000 | .98 | -0.002 | .25 |
| # years in ACO | -0.002 | .42 | -0.002 | .48 | -0.004 | .16 |
| PCMH*MU | 0.002 | .33 | 0.001 | .58 | 0.003 | .16 |
| PCMH*ACO | 0.003 | .29 | 0.003 | .34 | 0.004 | .21 |
| MU*ACO | -0.001 | .40 | -0.001 | .28 | 0.000 | .74 |
| All Three | 0.000 | .56 | -0.001 | .52 | -0.001 | .31 |

***Panel B***

| | **All Cause 30-day Readmissions** | | | | | |
|---|---|---|---|---|---|---|
| | **2017 with AHRF** | | **2017 without AHRF** | | **2017 >= 20 benes** | |
| | Coeff | P-Value | Coeff | P-Value | Coeff | P-Value |
| # years in PCMH | -0.036 | .23 | -0.065 | .02 | -0.057 | .05 |
| # years in MU | -0.001 | .69 | -0.004 | .18 | -0.002 | .46 |
| # years in ACO | -0.002 | .69 | 0.004 | .35 | -0.005 | .30 |
| PCMH*MU | 0.005 | .49 | 0.013 | .04 | 0.008 | .24 |
| PCMH*ACO | 0.018 | .12 | 0.025 | .02 | 0.030 | .008 |
| MU*ACO | 0.000 | .71 | 0.002 | .09 | -0.001 | .70 |
| All Three | -0.004 | .11 | -0.006 | .01 | -0.007 | .01 |

| ***Panel C*** | **All Cause ED Visits** | | | | | |
|---|---|---|---|---|---|---|
| | **2017 with AHRF** | | **2017 without AHRF** | | **2017 >= 20 benes** | |
| | Coeff | P-Value | Coeff | P-Value | Coeff | P-Value |
| # years in PCMH | -0.009 | .26 | -0.005 | .49 | -0.013 | .13 |
| # years in MU | 0.000 | .97 | 0.000 | .97 | -0.002 | .17 |
| # years in ACO | -0.001 | .75 | -0.001 | .83 | -0.003 | .25 |
| PCMH*MU | -0.001 | .60 | -0.001 | .38 | 0.000 | .92 |
| PCMH*ACO | 0.002 | .64 | 0.000 | .96 | 0.003 | .41 |
| MU*ACO | **-0.001** | **.01** | -0.002 | .009 | -0.001 | .09 |
| All Three | 0.000 | .66 | 0.000 | .97 | -0.001 | .40 |
| ***Panel D*** | | | | | | |

| | | | | | | |
|---|---|---|---|---|---|---|
| | | | | | | |
| | **Diabetes Guideline Adherence: Count – 4 Measures** | | | | | |
| | **2017 with AHRF** | | **2017 without AHRF** | | **2017 >= 20 benes** | |
| | Coeff | P-Value | Coeff | P-Value | Coeff | P-Value |
| # years in PCMH | **0.046** | **.01** | 0.042 | .02 | 0.053 | .01 |
| # years in MU | **0.022** | **<.001** | 0.021 | <.001 | 0.021 | .006 |
| # years in ACO | -0.005 | .70 | -0.006 | .61 | -0.008 | .58 |
| PCMH*MU | *-0.007* | *.10* | -0.006 | .16 | -0.008 | .08 |
| PCMH*ACO | -0.005 | .56 | -0.003 | .74 | -0.008 | .45 |
| MU*ACO | 0.000 | .93 | 0.001 | .82 | 0.001 | .82 |
| All Three | 0.001 | .67 | 0.000 | .84 | 0.001 | .56 |

| ***Panel E*** | **Annual Medicare Spending** | | | | | |
|---|---|---|---|---|---|---|
| | **2017 with AHRF** | | **2017 without AHRF** | | **2017 >= 20 benes** | |
| | Coeff | P-Value | Coeff | P-Value | Coeff | P-Value |
| # years in PCHM | 51.98 | .15 | 50.01 | .21 | 55.97 | .22 |
| # years in MU | 5.18 | .57 | 8.85 | .09 | 7.31 | .23 |
| # years in ACO | **-37.04** | **.01** | **-32.58** | **<.001** | **-33.69** | **<.001** |
| PCMH*MU | 3.48 | .69 | 4.30 | .62 | 3.86 | .69 |
| PCMH*ACO | -13.20 | .32 | -12.26 | .40 | -18.34 | .27 |
| MU*ACO | -2.03 | .58 | -1.49 | .52 | -1.45 | .57 |
| All Three | 1.69 | .55 | 1.36 | .67 | 1.70 | .64 |

© 2022 Adler-Milstein JR, et al. *JAMA Health Forum*

**eTable 7. One-Year versus Three-Year Marginal Effect Sizes**

| | One-year Marginal Effect Sizes | | | | | Three-year Marginal Effect Sizes | | | |
|---|---|---|---|---|---|---|---|---|---|
| **Admissions** | **Estimate** | **P-Value** | **Lower 95% CI** | **Upper 95% CI** | | **Estimate** | **P-Value** | **Lower 95% CI** | **Upper 95% CI** |
| PCMH Only: 1 Yr | -0.004 | .11 | -0.009 | 0.001 | 3 Yr | -0.012 | .10 | -0.027 | 0.002 |
| MU Only: 1 Yr | 0.000 | .93 | -0.001 | 0.001 | 3 Yr | 0.000 | .93 | -0.003 | 0.002 |
| ACO Only: 1 Yr | -0.001 | .42 | -0.002 | 0.001 | 3 Yr | -0.002 | .42 | -0.006 | 0.003 |
| PCMH and MU: 1 Yr | -0.004 | .09 | -0.008 | 0.000 | 3 Yr | -0.007 | .05 | -0.014 | 0.000 |
| PCMH and ACO: 1 Yr | -0.004 | .08 | -0.008 | 0.001 | 3 Yr | -0.006 | .32 | -0.019 | 0.006 |
| MU and ACO: 1 Yr | -0.001 | .31 | -0.002 | 0.001 | 3 Yr | -0.003 | .06 | -0.007 | 0.000 |
| All Three Programs: 1 Yr | -0.004 | .05 | -0.007 | 0.000 | 3 Yr | -0.006 | .06 | -0.012 | 0.000 |
| **Readmissions** | | | | | | | | | |
| PCMH Only: 1 Yr | -0.003 | .22 | -0.008 | 0.002 | 3 Yr | -0.009 | .21 | -0.024 | 0.005 |
| MU Only: 1 Yr | 0.000 | .69 | -0.001 | 0.000 | 3 Yr | 0.000 | .69 | -0.002 | 0.001 |
| ACO Only: 1 Yr | 0.000 | .69 | -0.001 | 0.001 | 3 Yr | 0.000 | .69 | -0.003 | 0.002 |
| PCMH and MU: 1 Yr | -0.003 | .17 | -0.007 | 0.001 | 3 Yr | -0.006 | .09 | -0.013 | 0.001 |
| PCMH and ACO: 1 Yr | -0.002 | .41 | -0.006 | 0.002 | 3 Yr | 0.004 | .57 | -0.010 | 0.018 |
| MU and ACO: 1 Yr | 0.000 | .49 | -0.001 | 0.001 | 3 Yr | -0.001 | .27 | -0.003 | 0.001 |
| All Three Programs: 1 Yr | -0.002 | .28 | -0.005 | 0.002 | 3 Yr | -0.003 | .43 | -0.009 | 0.004 |
| **ED Visits** | | | | | | | | | |
| PCMH Only: 1 Yr | -0.004 | .26 | -0.010 | 0.003 | 3 Yr | -0.011 | .25 | -0.030 | 0.008 |
| MU Only: 1 Yr | 0.000 | .97 | -0.001 | 0.001 | 3 Yr | 0.000 | .97 | -0.003 | 0.003 |
| ACO Only: 1 Yr | 0.000 | .75 | -0.002 | 0.002 | 3 Yr | -0.001 | .75 | -0.006 | 0.005 |
| PCMH and MU: 1 Yr | -0.004 | .14 | -0.010 | 0.001 | 3 Yr | **-0.014** | **.005** | -0.025 | -0.004 |
| PCMH and ACO: 1 Yr | -0.003 | .30 | -0.010 | 0.003 | 3 Yr | -0.006 | .65 | -0.033 | 0.021 |
| MU and ACO: 1 Yr | -0.001 | .36 | -0.003 | 0.001 | 3 Yr | **-0.006** | **.008** | -0.011 | -0.002 |
| All Three Programs: 1 Yr | -0.004 | .11 | -0.010 | 0.001 | 3 Yr | **-0.018** | **.006** | -0.031 | -0.005 |
| **Diabetes Guideline Adherence (Count)** | | | | | | | | | |
| PCMH Only: 1 Yr | **0.046** | **.01** | 0.010 | 0.082 | 3 Yr | **0.138** | **.01** | 0.030 | 0.246 |
| MU Only: 1 Yr | **0.022** | **<.001** | 0.010 | 0.034 | 3 Yr | **0.067** | **<.001** | 0.031 | 0.103 |
| ACO Only: 1 Yr | -0.005 | .70 | -0.029 | 0.019 | 3 Yr | -0.015 | .70 | -0.088 | 0.058 |
| PCMH and MU: 1 Yr | **0.061** | **<.001** | 0.028 | 0.095 | 3 Yr | **0.142** | **<.001** | 0.067 | 0.217 |
| PCMH and ACO: 1 Yr | 0.036 | .07 | -0.003 | 0.075 | 3 Yr | 0.077 | .31 | -0.072 | 0.225 |
| MU and ACO: 1 Yr | 0.018 | .19 | -0.008 | 0.044 | 3 Yr | 0.054 | .06 | -0.003 | 0.111 |
| All Three Programs: 1 Yr | **0.052** | **.006** | 0.015 | 0.090 | 3 Yr | **0.105** | **.02** | 0.015 | 0.194 |
| **Spending** | | | | | | | | | |
| PCMH Only: 1 Yr | 51 .98 | .15 | -19.00 | 122.95 | 3 Yr | 110.36 | .31 | -101.40 | 322.12 |
| MU Only: 1 Yr | 5.18 | .57 | -12.57 | 22.93 | 3 Yr | 37.66 | .16 | -15.26 | 90.57 |
| ACO Only: 1 Yr | **-37.04** | **.01** | -65.73 | -8.35 | 3 Yr | -84.39 | .06 | -170.49 | 1.71 |
| PCMH and MU: 1 Yr | 60.64 | .05 | -0.31 | 121.58 | 3 Yr | **208.59** | **.001** | 81.76 | 335.41 |
| PCMH and ACO: 1 Yr | 1.74 | .96 | -63.13 | 66.62 | 3 Yr | -65.29 | .54 | -273.55 | 142.97 |
| MU and ACO: 1 Yr | **-33.89** | **.04** | -65.79 | -1.99 | 3 Yr | -64.41 | .09 | -138.38 | 9.56 |
| All Three Programs: 1 Yr | 10.06 | .73 | -47.49 | 67.61 | 3 Yr | 43.03 | .48 | -76.22 | 162.28 |

© 2022 Adler-Milstein JR, et al. *JAMA Health Forum*

**eMethods**

**List of Ambulatory Care Sensitive Conditions**

We included in our measure of ACS admissions those specifically relevant to the Medicare population. They included:

PQI #1 Diabetes Short-Term Complications Admission

PQI #3 Diabetes Long-Term Complications Admission

PQI #5 Chronic Obstructive Pulmonary Disease (COPD) or Asthma in Older Adults Admission

PQI #8 Heart Failure Admission

PQI #11 Bacterial Pneumonia Admission

PQI #12 Urinary Tract Infection Admission

PQI #14 Uncontrolled Diabetes Admission

PQI #16 Lower-Extremity Amputation among Patients with Diabetes

We applied the ACS specifications as defined by AHRQ. In our sample, the mean number of ACS admissions per beneficiary per year was 0.037 (SD=0.25).

**Measuring Program Participation**

To measure PCMH participation, we used NPI-level data from NCQA on date of first recognition. The first year in which a clinician could receive recognition was 2009. In a given year, we classified an organization as participating in PCMH if more than half of the clinicians assigned to a given TIN had active PCMH recognition. As shown below, few organizations were near this threshold, such that assignment would not have changed at lower or higher thresholds. We used the annual Medicare Data on Provider Practice and Specialty (MD-PPAS) file that contains NPIs along with their TIN to link NPIs to TINs.

For MU participation, we used annual data on the NPIs of clinicians who attested along with their attestation date, beginning in the first year of the program (2011). Again, we used 50% as the cutoff for the TIN to be considered as participating in MU and few organizations were close to this cutoff. (See below for details). We also had to slightly adjust our approach to 2017 participation given program

© 2022 Adler-Milstein JR, et al. *JAMA Health Forum*

changes. Finally, we used annual data on TIN participation in the MSSP from CMS's Shared Savings Program Provider-level Research Identifiable File, which commenced in 2012. Detailed program participation data (and organizational demographics associated with different levels of participation) is reported in our prior work.[31]

**PCMH Participation (% NPIs within TIN)**

The graphs below plot the percent of NPIs within each TIN that had PCMH accreditation using 2016 data. We used this data to inform the decision about the cutoff for considering a TIN to be participating in PCMH in a given year. We selected 50% based on the fact that only 2,462 TINs were >0% and <100% out of 56,287 total TINs (4.3%). If we lowered threshold to 40%, only an additional 102 TINs would have been included. If we raised threshold to 60%, only an additional 56 TINs would have been included.

**For all TINs**

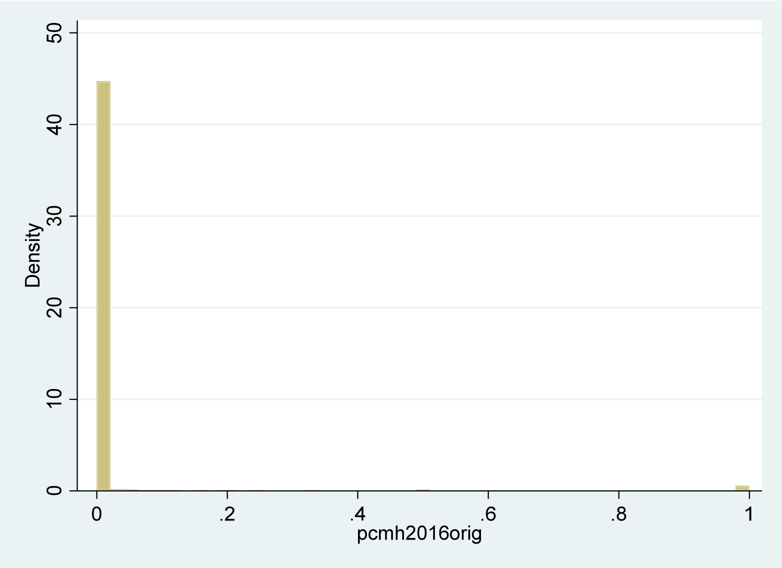

**For all TINs>0% and <100%**

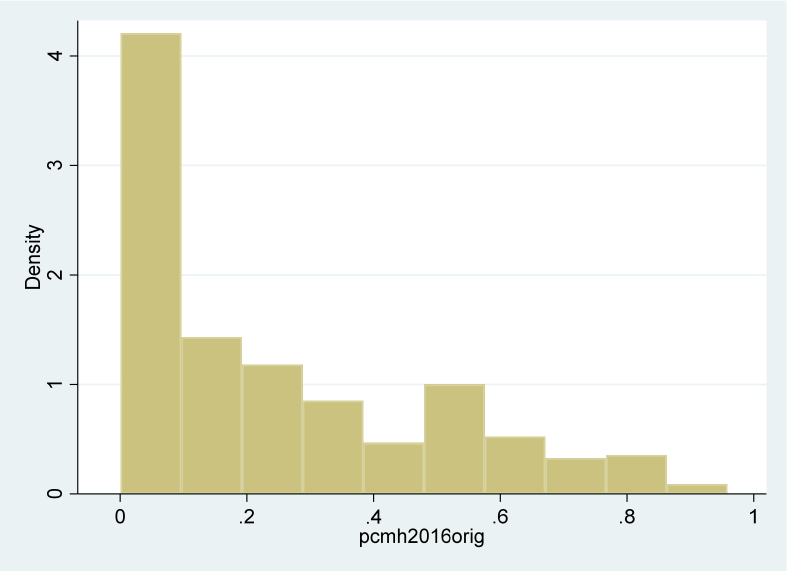


**MU Participation (% NPIs within TIN)**

The graphs below plot the percent of NPIs within each TIN that attested to MU using 2016 data. We used this data to inform the decision about the cutoff for considering a TIN to be participating in MU in a given year. We selected 50% based on the fact that 10,880 TINs were >0% and <100% out of 56,287 total TINs (19.3%). If we lowered threshold to 40%, an additional 1,050 TINs would have been included. If we raised threshold to 60%, an additional 790 TINs would have been included.

**For all TINs**

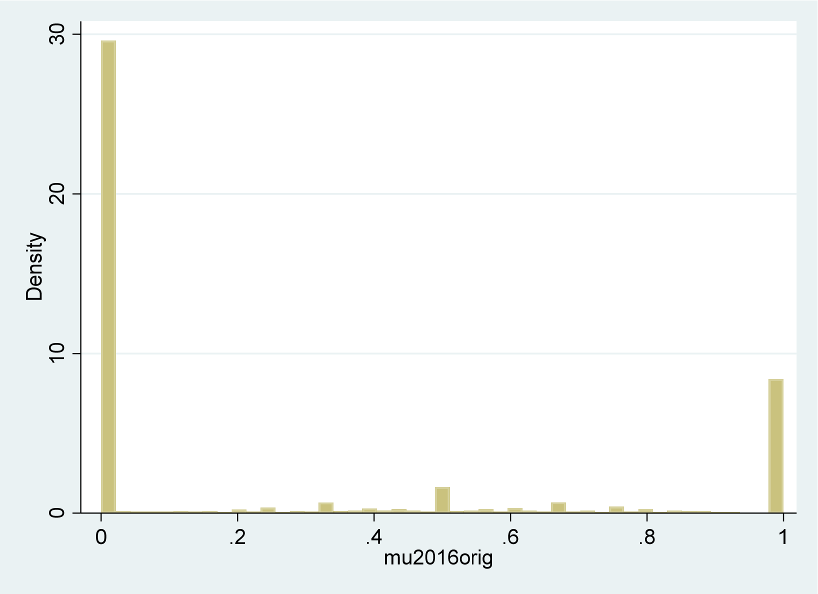

**For all TINs>0% and <100%**

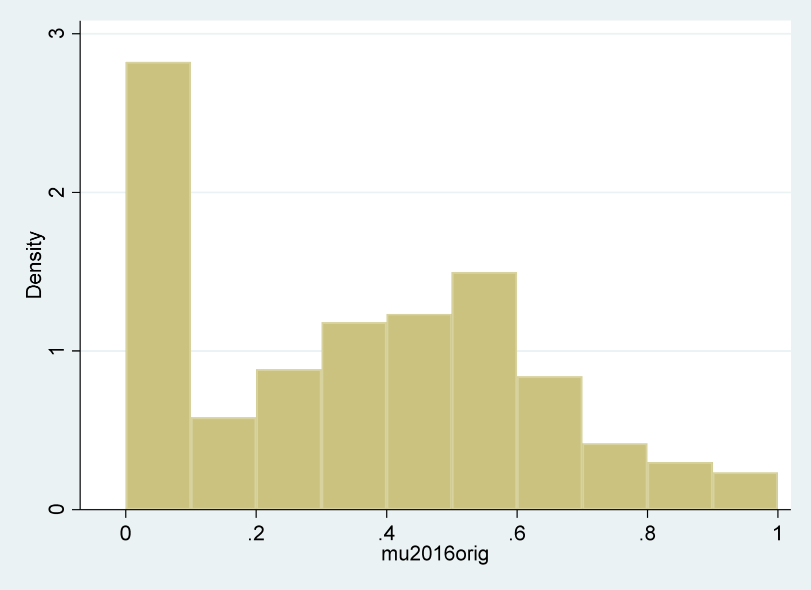


© 2022 Adler-Milstein JR, et al. *JAMA Health Forum*
